# Sociodemographic inequities and active transportation in adults from Latin America: an eight-country observational study

**DOI:** 10.1186/s12939-021-01524-0

**Published:** 2021-08-26

**Authors:** Gerson Ferrari, Juan Guzmán-Habinger, Javiera L. Chávez, André O. Werneck, Danilo R. Silva, Irina Kovalskys, Georgina Gómez, Attilio Rigotti, Lilia Yadira Cortés, Martha Cecilia Yépez García, Rossina G. Pareja, Marianella Herrera-Cuenca, Clemens Drenowatz, Carlos Cristi-Montero, Adilson Marques, Miguel Peralta, Ana Carolina B. Leme, Mauro Fisberg

**Affiliations:** 1 Escuela de Ciencias de la Actividad Física, el Deporte y la Salud, Universidad de Santiago de Chile (USACH), Las Sophoras 175, Estación Central, Santiago, Chile; 2 Especialidad medicina del deporte y la actividad física, Facultad de ciencias, Universidad Mayor, Santiago, Chile; 3 Datrics, Santiago, Chile; 4 Department of Nutrition, School of Public Health, Universidade de São Paulo (USP), São Paulo, Brazil; 5 Department of Physical Education, Federal University of Sergipe – UFS, São Cristóvão, Brazil; 6 Carrera de Nutrición, Facultad de Ciencias Médicas, Pontificia Universidad Católica Argentina, Buenos Aires, Argentina; 7 Departamento de Bioquímica, Escuela de Medicina, Universidad de Costa Rica, San José, Costa Rica; 8 Centro de Nutrición Molecular y Enfermedades Crónicas, Departamento de Nutrición, Diabetes y Metabolismo, Escuela de Medicina, Pontificia Universidad Católica, Santiago, Chile; 9 Departamento de Nutrición y Bioquímica, Pontificia Universidad Javeriana, Bogotá, Colombia; 10 Colégio de Ciencias de la Salud, Universidad San Francisco de Quito, Quito, Ecuador; 11 Instituto de Investigación Nutricional, Lima, Peru; 12 Centro de Estudios del Desarrollo, Universidad Central de Venezuela (CENDES-UCV)/Fundación Bengoa, Caracas, Venezuela; 13 Division of Sport, Physical Activity and Health, University of Education Upper Austria, 4020 Linz, Austria; 14 Physical Education School, IRyS Group, Pontificia Universidad Catolica de Valparaiso, Valparaiso, Chile; 15 Faculdade de Motricidade Humana, CIPER, Universidade de Lisboa, 1499-002 Lisbon, Portugal; 16 Faculdade de Medicina, ISAMB, Universidade de Lisboa, 1649-028 Lisbon, Portugal; 17 Centro de Excelencia em Nutrição e Dificuldades Alimentaes (CENDA) Instituto Pensi, Hospital Infantil Sabará, Fundação José Luiz Egydio Setubal, São Paulo, Brazil; 18 Departamento de Pediatria da Universidade Federal de São Paulo, São Paulo, Brazil; 19 Family Relations and Applied Nutrition, University of Guelph, Guelph, ON Canada

**Keywords:** Physical activity, Active commuting, Epidemiology, Sociodemographic, Equity, Latin America

## Abstract

**Background:**

Active transportation is a crucial sort of physical activity for developing sustainable environments and provides essential health benefits. This is particularly important in Latin American countries because they present the highest burden of non-communicable diseases relative to other worldwide regions. This study aimed to examine the patterns of active transportation and its association with sociodemographic inequities in Latin American countries.

**Methods:**

This cross-sectional study was conducted in eight countries. Participants (n = 8547, 18–65 years) self-reported their active transportation (walking, cycling, and total) using the International Physical Activity Questionnaire. Sex, age, ethnicity, socioeconomic level, education level, public and private transport use, and transport mode were used as sociodemographic inequities.

**Results:**

Participants spent a total of 19.9, 3.1, and 23.3 min/day with walking, cycling, and total active transportation, respectively. Mixed and other ethnicity (Asian, Indigenous, Gypsy, and other), high socioeconomic level as well as middle and high education level presented higher walking than Caucasian, low socioeconomic and education level. Private transport mode and use of ≥ 6 days/week of private transport showed lower walking than public transport mode and ≤ 2 days/week of private transport. Use of ≥ 3 days/week of public transport use presented higher walking than ≤ 2 days/week of public transport. Men had higher cycling for active transportation than women. Use of ≥ 3 days/week of public transport use presented higher cycling than ≤ 2 days/week of public transport. ≥6 days/week showed lower cycling than ≤ 2 days/week of private transport use. Men (*b*: 5.57: 95 %CI: 3.89;7.26), black (3.77: 0.23;7.31), mixed (3.20: 1.39;5.00) and other ethnicity (7.30: 2.55;12.04), had higher total active transportation than women and Caucasian. Private transport mode (-7.03: -11.65;-2.41) and ≥ 6 days/week of private transport use (-4.80: -6.91;-0.31) showed lower total active transportation than public transport mode and ≤ 2 days/week of private transport use. Use of 3–5 (5.10: 1.35;8.85) and ≥ 6 days/week (8.90: 3.07;14.73) of public transport use presented higher total active transportation than ≤ 2 days/week of public transport use. Differences among countries were observed.

**Conclusions:**

Sociodemographic inequities are associated differently with active transportation across Latin American countries. Interventions and policies that target the promotion of active policies transportation essential to consider sociodemographic inequities.

**Trial registration:**

ClinicalTrials.Gov NCT02226627. Retrospectively registered on August 27, 2014.

**Supplementary Information:**

The online version contains supplementary material available at 10.1186/s12939-021-01524-0.

## Introduction

More socioeconomically disadvantaged individuals have higher rates of cardiovascular disease (CVD), stroke, diabetes mellitus and cancer compared with more advantaged individuals [1]. Those living in socioeconomically disadvantaged situations also exhibit less healthy behaviours and worse health outcomes [2]. Transport in general is a key social factor of wellbeing, mainly in urban areas, which can help decrease social inequalities, improve people’s psychological and physical health, and decrease environmental pollution [3].

The Latin America region has undergone rapid urbanization with significant challenges in terms of transportation and urban planning. Besides, inequality is a major structural problem in Latin America. In the last decade, the region has experienced an increase in poverty rates, higher levels of unemployment, a deterioration in employment quality indicators, and stagnation in the decline of income inequality [4]. These trends are also of concern from the perspective of health inequalities since they can directly or indirectly undermine health and exacerbate the existing deep gaps in this area and Latin America is widely known as the world’s top unequal region [4]. Therefore, it is a region that is difficult to define and to understand [5–7]. Around 80 % of the Latin American population lives in urban areas at different levels of urbanization and different stages in the mobility transition [5]. In recent years, several attempts have been made to improve certain features of the urban environment and reduce social and spatial segregation against the marginalized population [8, 9].

Urban mobility, which is influenced by transport and land use planning, plays a direct role in the population’s health status through daily travel time and travel modes to their working or studying places [10]. As observed in the Latin America region, poor urban mobility leads to longer trips in daily transportation. In addition, two-thirds of daily commutes involve either sitting or standing in private or public transportation [10, 11]. Accordingly, initiatives targeting active transport have great potential to promote and sustain walking and cycling [5]. In this sense, transportation and urban planning are closely related to physical activity, free time, and sedentary behavior.

However, monitoring data on active transportation, particularly for walking and cycling, has been sparse in Latin America as these modes of transportation have been relegated to a secondary role in health and transportation research [12]. Failing to promote and monitor different levels of walking and cycling might jeopardize efforts supporting the agenda in the “Sustainable Development Goals” era. As has already been learned from the Millennium Development Goals experience, in which limitations arose related to a lack of data related to various criteria, including targets linked with sustainable healthy environments (e.g., Target C of Millennium Development Goals 7: “Ensure Environmental Sustainability”) [13]. Active transportation is a crucial physical activity component for developing sustainable environments as it provides health benefits and ancillary benefits related to greenhouse gas emissions. In addition to the global scale, this is mainly relevant for Latin American countries as they have the highest burden of non-communicable diseases relative to other regions [14], reinforcing historical health inequities.

Active transportation, such as walking or cycling, is associated with a reduced incidence of cardiovascular disease and mortality [15, 16]. Thus, it is an accessible and cheap alternative for more people to become physically active [17]. This type of physical activity can be easily integrated into everyday life, particularly for those not interested in engaging in other kinds of activities such as sports, training in gyms, etc. [18]. Increasing active transportation is a crucial population-based strategy, which aims to reverse the burden of CVD through decreasing physical inactivity [15, 16].

Research has shown that transport infrastructure can affect physical activity because transport systems can provide an incentive to use multimodal means of transport and increase physical activity in urban areas [19, 20]. Public transport can also contribute to the practice of physical activity because accessing transport services often requires walking [20]. Evidence also shows that car use and car dependence is a risk factor for obesity because excess driving leads to an imbalance between energy consumption and energy expenditure [19].

People who spend a significant amount of time sitting in traffic have been found to display a lower sense of community and higher social isolation [21, 22]. This is particularly problematic for Latin American cities (i.e., Bogotá, Cali, and Medellín [Colombia]; Quito [Ecuador]; Belo Horizonte and São Paulo [Brazil]; and Toluca, Mexico City, Guayaquil, and Guadalajara [Mexico]), where people spend a mean of 3.1 h/day in traffic [23]. Changes in transport and urban planning have to consider this complex perspective to achieve a better balance that favors active transport and reduces both non-active and total daily travel time while considering the urban environment of Latin American cities.

Due to the complexity and large number of associated and determinant factors of active transportation, evidence has shown that some population strata have greater opportunities for such practice. Previous studies have found that people who engage in active transportation have different sociodemographic characteristics than those who engage in leisure-time physical activity [24, 25]. Those studies, often performed in high-income countries, have shown the influence of sociodemographic variables, including age, sex, income, ethnic group, and education level, on walking or bicycling behavior [24, 26, 27]. To date, there is limited evidence on the associations of sociodemographic inequities with active transportation in low-middle income countries. A previous study analyzing data from six South American countries found that men and people with a lower educational level presented higher active transport [28]. Therefore, considering broader demographic inequities associated with active transportation should guide countries to direct interventions to those who need them the most. However, how different sociodemographic inequities are associated with active transportation in different modes (i.e., walking or cycling) in Latin American countries is still unknown. Therefore, this study aimed to examine the prevalence and the associations between sociodemographic inequities and the type of active transportation in adults from eight Latin American countries.

## Methods

### Design study

The Latin American Study of Nutrition and Health (Estudio Latinoamericano de Nutrición y Salud; ELANS) is a population-based multi-center study conducted in eight Latin American countries (Argentina, Brazil, Chile, Colombia, Costa Rica, Ecuador, Peru, and Venezuela) and aimed to investigate lifestyle behaviors and weight status of nationally representative samples from urban populations. Only urban location data were included to increase comparability across countries and reasons surrounding feasibility [29]. Data collection for ELANS took place from September 2014 to February 2015. All the study sites followed the standard protocol with all study personnel undergoing training, certification in the data collection, and all countries met local ethics requirements. The ELANS protocol is registered at ClinicalTrials.gov (#NCT02226627) and was approved by the Western Institutional Review Board (#20,140,605). Written informed consents were provided by all participants before their participation in the study.

### Sample

ELANS used a random complex multistage sample, designed to be representative in terms of geographical area, sex, age, and socioeconomic level, with a random selection of Primary Sampling Units (PSU) and Secondary Sampling Units (SSU) to recruit participants. Within each SSU, households were selected through systematic randomization and selection of respondents within a household using the birthday method (50 % of the sample chosen using the next birthday and 50 % using the last birthday), with quotas controlled for sex, age, and socioeconomic level. The sampling size required for sufficient precision was calculated with a 95 % confidence interval (95 % CI) and a maximum error of 3.5 %. Based on guidance from the U.S. National Center for Health Statistics [30], a study design effect of 1.75 was estimated to determine the minimum sample sizes required per strata (i.e., socioeconomic level, age, and sex) for each country. Sample weighting was computed for individual country level considering key variables of interest (the geographical area, sex, age, and socioeconomic level). Sample weighting was not applied on the unified (eight countries) database given the lack of an official publication of the Latin America urban population distribution. All analyses presented in the current paper were performed using this unified database. Details on study design and protocol have been previously published elsewhere [29, 31].

Participants were excluded from the study if at the recruitment they were pregnant/lactating, had a major physical/mental disease that influenced food intake and/or physical activity levels, were < 15 or > 65 years old, did not provide consent to participate in the study, or could not read.

A total sample of 9218 (n = 4409, 48.1 % men) participants aged between 15 and 65 was included in the ELANS study. The questionnaires were interviewer-administered during a home visit, and 8547 (18–65 years old) participants had complete data. We excluded adolescents (15 to 17 years old) from the analyses because the ELANS study did not include adolescents of all ages. Also, adolescents may have restricted independent mobility that may yield different active transportation than those observed in adults [32]. In addition, physical activity guidelines for adolescents differ from those for adults [33].

### Active transportation

Participants reported active transportation levels by completing the long-form of the last seven days interview version from the International Physical Activity Questionnaire (IPAQ) in Spanish and Portuguese languages [34]. We used only questions that covered the active transportation domain [31]. The IPAQ has been validated to assess physical activity in individuals aged 18–65 years in several countries [34].

For active transportation, the following questions were asked: (i) “Did you walk or ride a bicycle for at least 10 continuous minutes to move from one place to another?” (Yes, No); (ii) “On a typical week, how many days do you walk or ride a bicycle for at least 10 continuous minutes to move from one place to another?” and (iii) “On a typical day, how much time do you spend walking or riding a bicycle for transportation?” These questions were asked separately for walking and cycling. Time spent in active transportation was expressed as min/day and calculated in the same way as described for non-active transportation. Time (min/day) spent in each activity (i.e., walking and cycling) was calculated and used in the analysis. In this study, we used walking and cycling for transport separately and combined (total active transportation). Details on the assessment of walking and cycling for transport by IPAQ have been published elsewhere [31].

### Sociodemographic inequities

Sociodemographic inequities including sex, age, socioeconomic, education level, and ethnicity were assessed using standard questionnaires. Participants self-reported their age and were categorized into three age groups (18–30, 30–49, and 50–65 years) to obtain appropriate sample sizes. Due to different classification systems across countries, we used three levels of classification based on the national statistics used in each country and included equivalent characteristics for all countries. Socioeconomic level data was divided into three strata (low, medium, high) based on the national indexes used in each country [35]. A similar process to standardize the level of education was used: low (fundamental school), medium (high-school), and high (university degree) for all countries. Participants were asked about their ethnicity (Caucasian, black, mixed-race, and other [i.e., Asian, Indigenous, Gypsy, and other]) [29].

Participants were asked which transport mode they use in a typical week (i.e., bus, taxi, car, motorcycle, metro, other), and these responses were recategorized as public (i.e., bus and metro), private (i.e., taxi, car, motorcycle), and other. Furthermore, the frequency of public and private transport use (1 day, 2 days, 3 days, 4 days, 5 days; 6 days; or 7 days) was also assessed separately as potential correlates and was analysed as ≤ 2 days/week, 3–5 days/week, or ≥ 6 days/week.

### Statistical analysis

Participants who provided complete information for the variables of interest were analyzed in the present study. Kolmogorov Smirnov test was used to check the normal distribution of the data. As walking and cycling for transportation were not normally distributed, the descriptive statistics were presented as mean, median, and 95 % confidence intervals (95 %CI). Furthermore, frequencies, percentages, and values for the 25th and 75th percentile were also reported. For categorical analyses, Chi-square tests were applied. We used dichotomous outcome measures with “<10 continuous minutes” (no-active transportation) and “≥10 continuous minutes” (active transportation) for walking, cycling, and total active transportation because of the implications for public health [36]. Results were stratified by country, sex, age group, ethnicity, and socioeconomic and educational levels, transport mode, and public and private transport use.

Multilevel linear regression models, including region and cities as random effects, adjusted for country, sex, age, ethnicity, socioeconomic and education level, reporting unstandardized beta coefficients and 95 %CI were used to examine the associations between sociodemographic inequities with walking, cycling, and total active transportation. Data analyses were performed with SPSS (version 24 for Windows) (SPSS Inc., IBM Corp., Armonk, New York, NY, USA). We present the overall (i.e., pooled) and country-specific results (Additional file 1: Table S1-S3). A significance level of 5 % was adopted. The samples were weighted considering sociodemographic inequities, sex, and income for comparability sample within the population of each country [29].

## Results

Overall, 8547 participants aged 18–65 years old (mean: 37.4, 95 % CI: 37.1; 37.6) completed the questionnaire. On average, participants spent 19.9, 3.1, and 23.3 min/day in walking, cycling, and total active transportation, respectively. There were significant differences observed between countries, ethnicity, and public and private transport use for mean time spent walking for transportation; but no differences for sex, age, socioeconomic and education level, and transport mode. For cycling, there were significant differences observed between countries, sex, age group, ethnicity, education level, transport use, and public and private transport for mean time spent in cycling for transportation. For socioeconomic level, there were no significant differences. For total active transportation, significant differences were observed between countries, sex, ethnicity, education level, transport mode, and public and private transport use (Table 1).

**Table 1:** Characteristics of participants by sociodemographic inequities and the comparison with time spent in active transportation

Variables			Walking			Cycling			Total		
	N	%	Mean (95 %CI)of min/day	Median (25–75)of min/day	*p*^a^	Mean (95 %CI)of min/day	Median (25–75)of min/day	*p*^a^	Mean (95 %CI)of min/day	Median (25–75)of min/day	*p*^a^
Total	8547	100.0	19.9 (19.2; 20.6)	10.7 (0.0–25.0)		3.1 (2.7; 3.4)	0 (0–0)		23.3 (22.4; 24.1)	11.4 (0–30)	
Countries					< 0.001			< 0.001			< 0.001
Argentina	1177	13.8	20.4 (18.4; 22.4)	0 (0-25.7)		3.9 (2.8; 5.0)	0 (0–0)		24.5 (22.1; 26.9)	10 (0–30.0)	
Brazil	1872	21.9	17.1 (15.7; 18.5)	0 (0–20.0)		3.4 (2.6; 4.2)	0 (0–0)		20.9 (19.2; 22.5)	10 (0-25.7)	
Chile	811	9.5	17.8 (15.7; 19.8)	10.7 (0-21.4)		4.9 (3.3; 6.5)	0 (0–0)		23.0 (20.3; 25.6)	11.4 (0-25.7)	
Colombia	1154	13.5	21.3 (19.3; 23.3)	11.4 (0-25.7)		3.9 (2.8; 5.0)	0 (0–0)		25.5 (23.1; 27.9)	12.9 (0–30.0)	
Costa Rica	728	8.5	27.8 (24.9; 30.7)	14.3 (0-34.3)		5.0 (3.5; 6.5)	0 (0–0)		33.0 (29.7; 36.4)	17.1 (0–40.0)	
Ecuador	737	8.6	25.4 (22.9; 28.0)	15 (0–30.0)		1.6 (0.9; 2.3)	0 (0–0)		27.4 (24.7; 30.1)	17.1 (0–30.0)	
Peru	1018	11.9	21.4 (19.5; 23.2)	12.9 (0-28.6)		1.1 (0.6; 1.6)	0 (0–0)		22.7 (20.7; 24.6)	14.3 (0–30.0)	
Venezuela	1050	12.3	13.7 (12.0; 15.4)	0 (0-17.1)		0.8 (0.2; 1.4)	0 (0–0)		14.6 (12.8; 16.4)	0 (0–17.0 )	
Sex					0.159			< 0.001			0.001
Men	4016	47.0	20.4 (19.3; 21.5)	10 (0-21.4)		5.4 (4.7; 6.1)	0 (0–0)		26.2 (24.8; 27.6)	12.9 (0–30.0)	
Women	4531	53.0	19.5 (18.6; 20.4)	10.7 (0-25.7)		0.9 (0.7; 1.1)	0 (0–0)		20.5 (19.6; 21.5)	10.7 (0-25.7)	
Age group					0.705			0.002			0.149
18–30 years	4031	47.2	19.7 (18.6; 20.7)	10.7 (0-22.9)		3.4 (2.8; 4.0)	0 (0–0)		23.4 (22.1; 24.7)	12.9 (0–30.0)	
30–49 years	2626	30.7	20.1 (19.0; 21.2)	10 (0-25.7 )		2.5 (2.0; 3.0)	0 (0–0)		22.8 (21.6; 24.1)	10.7 (0–30.0)	
50–65 years	1890	22.1	20.1 (18.5; 21.7)	10.7 (0-25.7)		3.5 (2.5; 4.4)	0 (0–0)		23.8 (21.8; 25.8)	11.4 (0–30.0)	
Ethnicity					< 0.001			0.044			< 0.001
Caucasian	2979	34.9	18.0 (16.9; 19.2)	0 (0-21.4)		2.9 (2.4; 3.5)	0 (0–0)		21.3 (19.9; 22.6)	10 (0-25.7)	
Black	547	6.4	20.1 (17.2; 23.0)	10.7 (0-21.4)		4.3 (2.5; 6.1)	0 (0–0)		24.6 (20.9; 28.2)	11.4 (0–30.0)	
Mixed	3917	45.8	21.1 (20.1; 22.1)	11.4 (0-25.7)		2.9 (2.4; 3.4)	0 (0–0)		24.3 (23.1; 25.4)	12.9 (0–30.0)	
Other	1104	12.9	23.2 (19.2; 27.1)	12.9 (0–30.0)		4.8 (2.1; 7.5)	0 (0–0)		28.3 (23.5; 33.1)	15 (0-34.3)	
Socioeconomic level					0.332			0.592			0.569
Low	4442	52.0	19.6 (18.6; 20.5)	11.4 (0-25.7)		3.2 (2.7; 3.7)	0 (0–0)		23.1 (22.0; 24.2)	12.9 (0–30.0)	
Middle	3291	38.5	20.2 (19.1; 21.4)	10.7 (0–25.0)		2.9 (2.4; 3.4)	0 (0–0)		23.4 (22.0; 24.7)	11.4 (0–30.0)	
High	814	9.5	20.6 (18.4; 22.9)	10 (0-22.9 )		2.8 (1.7; 3.9)	0 (0–0)		23.7 (21.1; 26.3)	11.4 (0-28.6)	
Education level					0.382			0.001			0.040
Low	5049	59.1	20.7 (19.7; 21.6)	10.7 (0-25.7)		3.6 (3.1; 4.1)	0 (0–0)		24.5 (23.4; 25.6)	12.1 (0-25.7)	
Middle	2635	30.8	18.8 (17.6; 20.0)	10.7 (0-21.4)		2.2 (1.7; 2.8)	0 (0–0)		21.3 (20.0; 22.6)	11.4 (0-21.4)	
High	863	10.1	18.7 (16.5; 20.9)	10 (0-21.4)		2.3 (1.3; 3.3)	0 (0–0)		21.1 (18.6; 23.6)	10.7 (0-21.4)	
Transport mode											
Public	4904	57.4	21.3 (20.4; 22.1)	11.4 (2.8–25.7)	0.055	2.0 (1.7; 2.3)	0 (0–0)	0.001	23.3 (22.4; 24.3)	12.9 (4.3–28.6)	0.001
Private	2198	25.7	19.3 (17.9; 20.7)	8.6 (0–20.0)		2.2 (1.7; 2.8)	0 (0–0)		21.5 (19.8; 23.2)	8.6 (0-21.4)	
Other	1445	16.9	21.2 (19.5; 23.3)	8.6 (0–25.0)		7.9 (6.6; 9.4)	0 (0–0)		29.2 (26.8; 31.7)	12.0 (0–30.0)	
Public transport use											
≤ 2 days/week	3424	40.1	20.4 (19.3; 21.6)	8.6 (0-21.4)	0.001	3.8 (3.2; 4.4)	0 (0–0)	0.001	24.2 (22.7; 25.5)	10.0 (0-28.6)	0.001
3–5 days/week	2288	26.8	18.9 (17.8; 20.1)	10.7 (3.5–21.4)		1.9 (1.4; 2.4)	0 (0–0)		20.8 (19.5; 22.0)	11.4 (4.3–51.5)	
≥ 6 days/week	2835	33.2	22.8 (21.5; 24.2)	11.4 (1.7–25.7)		3.2 (2.6; 3.8)	0 (0–0)		25.9 (24.5; 27.4)	12.8 (2.8–30.0)	
Private transport use											
≤ 2 days/week	4831	56.5	22.8 (21.8; 23.7)	11.4 (2.8–25.7)	0.001	3.7 (3.2; 4.2)	0 (0–0)	0.001	26.5 (25.4; 27.7)	12.9 (4.3; 30.0)	0.001
3–5 days/week	970	11.3	20.2 (18.2; 22.2)	10.0 (1.4–21.4)		1.6 (1.0; 2.2)	0 (0–0)		21.8 (19.5; 23.9)	10.7 (2.9–22.9)	
≥ 6 days/week	2746	32.1	17.5 (16.2; 18.6)	7.1 (0–20.0)		2.5 (1.9; 3.0)	0 (0–0)		19.9 (18.7; 21.4)	8.6 (0-21.4)	

^a^ Mann-Whitney or kruskal-Wallis test for the comparison of means; other ethnicity (Asian, Indigenous, Gypsy, and other)

Total no-active transportation was 44.2 % of the participants (46.8 and 94 % of walking and cycling, respectively). The country with the highest proportion of non-transportation walking was Venezuela (60.3 %), and the lowest was Ecuador (30.2 %). Regarding non-active cycling transportation, Venezuela was the country with the highest percentage (98.5 %), while Costa Rica was the country with the lowest percentage of non-cycling users (89,6 %). The highest proportion of total non-active transportation was Venezuela (59 %), and the lowest was Ecuador (28.6 %). The prevalence of countries not using cycling for active transportation was higher than those who report walking for the total sample, sex, age group, ethnicity, socioeconomic and education level, transport mode, and public and private transport use (Table 2).

**Table 2:** Sociodemographic inequities (%; 95 %CI) of the population according to no-active transportation (< 10 continuous minutes)

Variables	Walking	Cycling	Total
Total	46.8 (45.7; 47.9)	94.0 (93.6; 94.5)	44.2 (43.1; 45.3)
Countries			
Argentina	52.1 (49.2; 55.0)	92.6 (91.3; 94.0)	48.7 (45.8; 51.6)
Brazil	50.7 (48.5; 53.1)	93.2 (92.2; 94.3)	47.8 (45.5; 50.2)
Chile	47.2 (43.8; 50.7)	91.6 (89.9; 93.4)	43.2 (39.8; 46.7)
Colombia	44.4 (41.5; 47.3)	93.4 (92.1; 94.8)	42.2 (39.4; 45.2)
Costa Rica	39.3 (35.9; 43.0)	89.6 (87.6; 91.7 )	34.8 (31.5; 38.3)
Ecuador	30.2 (26.9; 33.6)	95.9 (94.6; 97.2)	28.6 (25.4; 31.9)
Peru	39.5 (36.5; 42.5)	96.8 (95.9; 97.8)	38.1 (35.2; 41.2)
Venezuela	60.3 (57.3; 63.3)	98.5 (97.9; 99.2)	59.0 (56.0; 62.1)
Sex			
Men	47.6 (46.1; 49.2)	90.3 (89.4; 91.1)	43.3 (41.8; 44.9)
Women	46.1 (40.8; 54.0)	97.5 (97.0; 97.9)	45.0 (43.5; 46.5)
Age group			
18–30 years	45.3 (43.5; 47.0)	92.9 (92.1; 93.7)	42.0 (40.3; 43.7)
30–49 years	48.2 (46.5; 49.9)	94.7 (94.1; 95.4)	46.0 (44.3; 47.7)
50–65 years	47.1 (44.7; 49.5)	94.7 (93.7; 95.6)	44.8 (42.5; 47.2)
Ethnicity			
Caucasian	52.1 (50.3; 53.9)	93.9 (93.1; 94.7)	49.2 (47.4; 51.0)
Black	46.4 (42.3; 50.7)	92.4 (90.5; 94.6)	43.2 (39.0; 47.4)
Mixed	43.6 (42.1; 45.1)	94.6 (94.0; 95.2)	41.2 (39.7; 42.7)
Other	40.5 (35.0; 46.5)	91.7 (89.0; 94.8)	37.9 (32.4; 43.8)
Socioeconomic level			
Low	45.7 (42.3; 49.2)	94.8 (93.4; 96.2)	43.1 (39.7; 46.6)
Middle	46.3 (44.6; 48.1)	93.8 (93.1; 94.6)	43.7 (42.0; 45.5)
High	47.4 (45.9; 48.9)	94.0 (93.4; 94.7)	44.7 (43.2; 46.2)
Education level			
Low	46.6 (45.2; 48.0)	93.1 (92.5; 93.8)	47.6 (38.8; 58.2)
Middle	46.5 (44.6; 48.6)	95.5 (94.8; 96.3)	43.4 (42.0; 44.8)
High	49.0 (45.6; 52.5)	95.1 (93.8; 96.5)	44.8 (42.9; 46.8)
Transport mode			
Public	43.6 (42.2; 45.0)	95.3 (94.7; 95.8)	41.5 (40.1; 42.9)
Private	52.9 (50.8; 55.1)	95.6 (94.8; 96.4)	50.6 (48.4; 52.8)
Other	45.8 (42.7; 49.0)	82.3 (80.0; 84.6)	38.8 (35.8; 41.9)
Public transport use			
≤ 2 days/week	51.5 (49.8; 53.2)	92.9 (92.1; 93.7)	48.4 (46.7; 50.1)
3–5 days/week	44.8 (42.8; 46.9)	95.5 (94.8; 96.3)	42.7 (40.7; 44.8)
≥ 6 days/week	42.6 (40.8; 44.4)	94.1 (93.3; 94.9)	40.1 (38.3; 42.0)
Private transport use			
≤ 2 days/week	43.5 (42.1; 44.9)	93.2 (92.5; 93.8)	40.5 (39.1; 41.9)
3–5 days/week	44.8 (41.7; 47.9)	95.9 (94.9; 97.1)	42.6 (39.6; 45.8)
≥ 6 days/week	44.8 (41.7; 47.9)	94.8 (94.0; 95.5)	51.3 (49.4; 53.3)

Other ethnicity (Asian, Indigenous, Gypsy, and other)

Table 3 shows the results of the multilevel linear regression models for the effects of sociodemographic inequities on walking for active transportation. In the overall sample, mixed-race (3.3 min/day) and other ethnicities (5.4 min/day) presented higher walking for active transportation than Caucasians. High (-3.5 min/day) and middle- (-2.5 min/day) education levels presented lower walking for active transportation than low education level. Overall, private transport mode (-9.1 min/day) and use of ≥ 6 days/week (-3.6 min/day) of private transport showed lower walking for active transportation than public transport mode and ≤ 2 days/week of private transport use. On the other hand, use of 3–5 days/week (7.0 min/day) and ≥ 6 days/week (13.6 min/day) of public transport use presented higher walking for active transportation than ≤ 2 days/week of public transport use (Table 3).

**Table 3:** Multilevel linear regression models (*b* coefficient (95 %CI)) between sociodemographic inequities and walking for active transportation

Sociodemographic inequities	Overall
Sex ^a^	
Women	1
Men	0.79 (-0.66; 2.24)
Age group ^b^	
50–65 years	1
30–49 years	-0.01 (-1.98; 1.96)
18–30 years	-0.58 (-2.56; 1.41)
Ethnicity ^c^	
Caucasian	1
Black	2.47 (-0.57; 5.51)
Mixed	3.30 (1.74; 4.85)
Other	5.37 (1.29; 9.45)
Socioeconomic level ^d^	
Low	1
Middle	1.50 (-0.09; 3.09)
High	2.82 (0.10; 5.54)
Education level ^e^	
Low	1
Middle	-2.45 (-4.14; -0.76)
High	-3.49 (-6.19; -0.79)
Transport mode ^f^	
Public	1
Private	-9.13 (-15.11; -3.15)
Other	4.85 (-1.09; 10.80)
Public transport use ^f^	
≤ 2 days/week	1
3–5 days/week	7.06 (2.45; 11.67)
≥ 6 days/week	13.64 (5.57; 21.71)
Private transport use ^f^	
≤ 2 days/week	1
–5 days/week	-2.60 (-5.90; 1.30)
≥ 6 days/week	-3.61 (-6.91; -0.31)

Multilevel linear regression models, including region and cities as random effects:

^a﻿^ Adjustment: country, age, ethnicity, socioeconomic and education level;

^b^ Adjustment: country, sex, ethnicity, socioeconomic and education level;

^c^ Adjustment: country, sex, age, socioeconomic and education level;

^d^ Adjustment: country, sex, age, ethnicity, and education level;

^e^ Adjustment: country, sex, age, ethnicity, and socioeconomic level;

^f^ Adjustment: country, sex, age, ethnicity, socioeconomic, and education level;

*CI* confidence interval

Other ethnicity (Asian, Indigenous, Gypsy, and other)

The country-specific results of associations of total and type of active transportation with sociodemographic inequities are available in Tables S1, S2, S3. Different associations by country were observed between active transportation and sociodemographic inequities. Men from Brazil and Ecuador, black ethnicity from Argentina and Brazil, mixed-race and other ethnicities from Chile presented higher walking for active transportation than women and Caucasian ethnicities. Private transport mode from Colombia and ≥ 6 days/week of private transport use from Brazil and Colombia presented lower walking for active transportation than public transport mode and ≤ 2 days/week of public transport use. The use of public transport use (3–5 and ≥ 6 days/week) from Costa Rica, Ecuador, and Venezuela were higher than ≤ 2 days/week of public transport use (Table S1).

For the overall sample, men (4.5 min/day) had higher cycling for active transportation than women. The middle education level presented lower cycling for active transportation than the low education level. Reporting 3–5 (4.1 min/day) and ≥ 6 days/week (7.4 min/day) of public transport use was associated with higher cycling for active transportation compared to ≤ 2 days/week of public transport use. Private transport use (≥ 6 days/week; -5.6 min/day) was associated with lower cycling for active transportation than ≤ 2 days/week of private transport use (Table 4).

**Table 4:** Multilevel linear regression models (*b* coefficient (95 % CI)) between sociodemographic inequities and cycling for active transportation by country

Sociodemographic inequities	Overall
Sex ^a^	
Women	1
Men	4.45 (3.71; 5.19)
Age group ^b^	
50–65 years	1
30–49 years	-0.86 (-1.86; 0.14)
18–30 years	-0.38 (-1.39; 0.63)
Ethnicity ^c^	
Caucasian	1
Black	1.42 (-0.13; 2.97)
Mixed	-0.10 (-0.89; 0.69)
Other	1.85 (-0.21; 3.92)
Socioeconomic level ^d^	
Low	1
Middle	-0.14 (-0.95; 0.67)
High	0.23 (-1.15; 1.61)
Education level ^e^	
Low	1
Middle	-1.28 (-2.14; -0.42)
High	-1.25 (-2.61; 0.12)
Transport mode ^f^	
Public	1
Private	-2.28 (-5.14; 0.58)
Other	-1.20 (-3.61; 1.20)
Public transport use^f^	
≤ 2 days/week	1
3–5 days/week	4.10 (2.50; 5.70)
≥ 6 days/week	7.42 (3.49; 11.35)
Private transport use^f^	
≤ 2 days/week	1
3–5 days/week	-4.62 (-7.91; 3.31)
≥ 6 days/week	-5.60 (-6.91; -4.30)

Multilevel linear regression models, including region and cities as random effects:

^a^ Adjustment: country, age, ethnicity, socioeconomic and education level;

^b^ Adjustment: country, sex, ethnicity, socioeconomic and education level;

^c^ Adjustment: country, sex, age, socioeconomic and education level;

^d^ Adjustment: country, sex, age, ethnicity, and education level;

^e^ Adjustment: country, sex, age, ethnicity, and socioeconomic level;

^f^ Adjustment: country, sex, age, ethnicity, socioeconomic, and education level;

*CI* confidence interval

Other ethnicity (Asian, Indigenous, Gypsy, and other)

Men from all countries, black race from Argentina, and mixed-race from Brazil had higher cycling for active transportation than women and Caucasians. Middle education level from Brazil presented lower cycling for active transportation compared to participants with low education level. Another transport mode from Argentina, Brazil, Chile, Colombia, and Costa Rica and ≥ 6 days/week from Colombia of public transport use presented higher cycling for active transportation than public transport mode and ≤ 2 days/week of public transport use. Private transport use of 3–5 and ≥ 6 days/week from Brazil and ≥ 6 days/week from Chile was lower than ≤ 2 days/week (Table S2).

In the overall sample, men (5.6 min/day), black-race (3.8 min/day), mixed-race (3.2 min/day), and other ethnicities (7.3 min/day) had higher total active transportation than women and Caucasian, respectively. Additionally, higher (-4.8 min/day) and middle (-3.8 min/day) education levels presented lower levels of active transportation than participants with low education level. Private transport mode (-7.0 min/day) and ≥ 6 days/week of private transport use (-4.8 min/day) was associated with lower total active transportation than public transport mode and ≤ 2 days/week of private transport use. Use of 3–5 days/week (5.1 min/day) and ≥ 6 days/week (8.9 min/day) of public transport use presented higher total active transportation than ≤ 2 days/week of public transport use (Table 5).

**Table 5:** Multilevel linear regression models (*b* coefficient (95 % CI)) between sociodemographic inequities and total active transportation by country

Sociodemographic inequities	Overall
Sex ^a^	
Women	1
Men	5.57 (3.89; 7.26)
Age group ^b^	
50–65 years	1
30–49 years	-0.91 (-3.21; 1.38)
18–30 years	-0.82 (-3.14; 1.49)
Ethnicity ^c^	
Caucasian	1
Black	3.77 (0.23; 7.31)
Mixed	3.20 (1.39; 5.00)
Other	7.30 (2.55; 12.04)
Socioeconomic level ^d^	
Low	1
Middle	1.26 (-0.59; 3.12)
High	3.00 (-0.15; 6.16)
Education level ^e^	
Low	1
Middle	-3.80 (-5.77; -1.83)
High	-4.83 (-7.96; -1.69)
Transport mode ^f^	
Public	1
Private	-7.03 (-11.65; -2.41)
Other	0.70 (-3.50; 4.20)
Public transport use ^f^	
≤ 2 days/week	1
3–5 days/week	5.10 (1.35; 8.85)
≥ 6 days/week	8.90 (3.07; 14.73)
Private transport use ^f^	
≤ 2 days/week	1
3–5 days/week	-3.31 (-6.16; 0.46)
≥ 6 days/week	-4.80 (-6.91; -0.31)

Multilevel linear regression models, including region and cities as random effects:

^a^ Adjustment: country, age, ethnicity, socioeconomic and education level;

^b^ Adjustment: country, sex, ethnicity, socioeconomic and education level;

^c^ Adjustment: country, sex, age, socioeconomic and education level;

^d^ Adjustment: country, sex, age, ethnicity, and education level;

^e^ Adjustment: country, sex, age, ethnicity, and socioeconomic level;

^f^ Adjustment: country, sex, age, ethnicity, socioeconomic, and education level;

*CI* confidence interval

Other ethnicity (Asian, Indigenous, Gypsy, and other)

Total active transportation was higher in men (Brazil, Chile, Colombia and, Ecuador) compared with women regardless of age, ethnicity, socioeconomic and education level. Black-race from Argentina and Brazil, mixed-races from Brazil, and Chile, and other ethnicities from Brazil, and Chile had higher walking for active transportation than Caucasian ethnicities. Private transport use (3–5 days/week and ≥ 6 days/week) from Brazil, Chile, and Colombia was lower than ≤ 2 days/week of private transport use (Table S3).

## Discussion

This study aimed to examine the patterns of active transportation and the association with sociodemographic inequities in adults from eight Latin American countries. We found that a lower education level, being of other ethnicity and being male, were associated with higher levels of total active transportation. Furthermore, private transport mode and ≥ 6 days/week of private transport use were associated with lower total active transportation compared to public transport mode and ≤ 2 days/week of private transport use. Specifically, participants with higher education levels and ≤ 2 days/week of private transport mode presented reduced time on walking and cycling, while men showed increased levels for cycling but not for walking transportation. The use of public transport for 3–5 days/week and ≥ 6 days/week was associated with higher total active transportation compared to ≤ 2 days/week of public transport use. Participants from other ethnicities than Caucasians showed increased levels for walking, but not cycling.

The results according to sex in cycling and total active transportation show clear inequality, since men had higher levels than women. However, the current study did not detect any sex inequalities in walking for active transportation. Women are more likely to make accompanied and versatile trips that require carrying various things, which are all less suited for cycling (and more suited for walking) [37]. The sex gap in cycling against women has been constantly detected in other places without a robust cycling culture [37, 38]. It might also be related to infrastructural preferences and cultural norms, including greater risk aversion among women [37], out-group stereotypes, and experiences of marginalization [38]. In addition, safety-related issues may explain this relationship; thus, women may feel less secure in pedaling in areas of heavy traffic and higher crime rate [39, 40]. This has important implications for women’s health, as there are higher levels of physical inactivity in this group [41]. A higher level of physical inactivity is detrimental to physical and mental health, including higher levels of overweight and obesity [42, 43]. Given that physical and mental health implies higher health expenses and generates higher levels of disability, this would place women at disadvantage [44]. Taking these factors together, it implies another barrier for gender equality in Latin America. As shown by Heise et al. “gender inequality and restrictive gender norms are powerful determinants of health and wellbeing” [45].

Education level has an inverse association with physical activity level in adults from South American countries [28]. Nonetheless, the majority of the studies from high-income countries have found a positive association between education level and active transportation [46] the evidence suggests variability in low- and middle-income countries due to different factors, for example, in low- and middle-income countries there is a lower availability of individual motor vehicles [47]. Therefore the inequalities in access to individual transportation modes might be increased compared to those in high-income countries. Suggesting that active transportation is a need for several workers with low education level [48]. Additionally, using public transportation is associated with increased physical activity, as public transportation users have to walk to a bus/train station [49, 50].

Although we found that being of other ethnicity was associated with higher levels of active transportation as these people walked more for transportation. The association between ethnicity and higher walking for transport can be a residual of both the lower socioeconomic and lower education levels among the non-white population due to the long-term history of disadvantages and segregation in Latin America. Even when differences in education level and socioeconomic status are considered, the proportion of non-white people living in disadvantaged and deprived areas is higher and, therefore, the access to private vehicles is lower.

In general, we found that the use of private transport mode and ≥ 6 days/week of private transport were associated with lower total active transportation. Therefore, facilitating public transport and safe opportunities for walking and cycling could be a viable strategy for municipalities to enhance the health and well-being of their population. At the local level, an acceptable urban proposal will only be fully effective if it is reinforced by well-implemented city-wide and region-wide combined policies that generate available employment, education, facilities, and high-quality public transport [51]. Several Latin American countries have advanced in infrastructure to encourage the use of active transportation, being the case of some cities such as Bogotá in Colombia, which invested in creating exclusive bike paths [42, 52]. The patterns of mode of transportation among the different socioeconomic levels and other social conditions in Latin America are closely related with the urban processes of the region. These processes have been associated with a prevalence of social and environmental inequalities, unplanned and disorganized growth, and underlying convergence of political and socioeconomic indicators [52].

Potential interventions aiming to increase active transportation should also consider variations in sociodemographic inequities and focus on groups with lower active transportation levels, including women and individuals with higher education levels. Future interventions should emphasize integrating transportation systems that reduce barriers and encourage active transportation [53, 54]. In addition, local differences between countries need to be considered. For example, all the ELANS countries showed variations in cycling transportation by sex, but only Brazil and Ecuador showed variations in walking for transport. Brazil and Colombia were the countries with the largest variations in active transportation according to socioeconomic status and education levels. At the same time, only Argentina, Brazil, and Chile presented variations for walking for transport according to ethnicity.

Our study shed light on the association of different sociodemographic inequities with different modes of active transportation (i.e., walking and cycling), using a representative sample from eight Latin American countries. However, some limitations should be taken into account. The cross-sectional nature of this type of study limited our ability to assess causality and there may even be the possibility of reverse causality. Despite the adjustment for several confounding factors, the effect of other vital covariates that were not measured in the ELANS study could not be ruled out, which might explain some of the associations found between active transportation and sociodemographic inequities. Additionally, the self-reported nature of active transportation and traffic data may be prone to misreporting bias, obscuring the true nature and magnitude of associations found. Large-scale studies with device-measured active transportation (e.g., GPS equipment), however, remain limited, especially in low- and middle-income countries [55, 56].

## Conclusions

Findings from this cross-national study showed considerable variation in levels of walking and cycling for active transportation according to sex, ethnicity, education levels, public and private transport use, and transport mode across eight Latin American countries. The different associations between countries have implications for future research and may inform public health policies and behavioral-change strategies at local and national level. Intervention studies are needed to increase active transportation in different domains. Future studies should investigate potential mediators linking the variation in different sociodemographic inequities and active transportation.

The authors would like to thank the staff and participants from each of the participating sites who made substantial contributions to ELANS. The following are members of ELANS Study Group: Chairs: Mauro Fisberg and Irina Kovalskys; Co-chair: Georgina Gómez Salas; Core Group Members: Attilio Rigotti, Lilia Yadira Cortés Sanabria, Georgina Gómez Salas, Martha Cecilia Yépez García, Rossina Gabriella Pareja Torres and Marianella Herrera-Cuenca; Steering committee: Berthold Koletzko, Luis A. Moreno and Michael Pratt; Accelerometry analysis: Priscila Bezerra Gonçalves, and Claudia Alberico; Physical activity advisor: Gerson Ferrari. Nutrition Advisors: Regina Mara Fisberg and Agatha Nogueira Previdelli. Project Managers: Viviana Guajardo, and Ioná Zalcman Zimberg.

## Supplementary Information

**Additional file 1:**

## Data Availability

The datasets generated and/or analyzed during the current study are not publicly available due the terms of consent/assent to which the participants agreed but are available from the corresponding author on reasonable request. Please contact the corresponding author to discuss availability of data and materials.
