# Supplementary material for: Sociodemographic inequities and active transportation in adults from Latin America: an eight-country observational study
Source: Int J Equity Health. 2021 Aug 26;20:190. doi: 10.1186/s12939-021-01524-0 (PMC8390191; doi:10.1186/s12939-021-01524-0)
Supplement: Supplementary file 1 — Additional file 1: [file 12939_2021_1524_MOESM1_ESM.doc]

**Table S1** Multilevel linear regression models (*b* coefficient (95%CI)) between sociodemographic inequities and walking for active transportation by country.

| Sociodemographic  inequities | Argentina | | Brazil | | Chile | | Colombia | | Costa Rica | | Ecuador | | Peru | | Venezuela | |  |
| --- | --- | --- | --- | --- | --- | --- | --- | --- | --- | --- | --- | --- | --- | --- | --- | --- | --- |
| Sex ^1^ |  | |  | |  | |  | |  | |  | |  | |  | |  |
| Women | 1 | | 1 | | 1 | | 1 | | 1 | | 1 | | 1 | | 1 | |  |
| Men | -4.20 (-8.40; 0.00) | | 3.12 (0.25; 5.98) | | 3.64 (-0.81; 8.09) | | 2.42 (-1.85; 6.69) | | -0.41 (-6.49; 5.66) | | 5.61 (0.45; 10.76) | | 0.10 (-3.75; 3.94) | | -3.39 (-6.81; 0.02) | |  |
| Age group ^2^ |  | |  | |  | |  | |  | |  | |  | |  | |  |
| 50-65 years | 1 | | 1 | | 1 | | 1 | | 1 | | 1 | | 1 | | 1 | |  |
| 30-49 years | -4.22 (-9.59; 1.15) | | 0.64 (-3.19; 4.46) | | -1.20 (-7.12; 4.72) | | 1.82 (-3.83; 7.46) | | 9.14 (0.73; 17.56) | | -3.69 (-11.18; 3.79) | | -1.73 ( -7.28; 3.89) | | -0.48 (-5.34; 4.38) | |  |
| 18-30 years | -3.45 (-9.01; 2.12) | | -0.09 (-4.10; 3.92) | | 1.93 (-4.00; 7.86) | | -0.23 (-5.86; 5.40) | | 7.71 (-0.70; 16.12) | | -8.15 (-15.64; -0.66) | | -3.35 (-8.79; 2.09) | | 1.40 (-3.42; 6.21) | |  |
| Ethnicity ^3^ |  | |  | |  | |  | |  | |  | |  | |  | |  |
| Caucasian | 1 | | 1 | | 1 | | 1 | | 1 | | 1 | | 1 | | 1 | |  |
| Black | 102.5 (31.5; 173.5) | | 4.59 (0.75; 8.44) | | - | | 3.00 (-5.57; 11.6) | | 0.11 (-21.78; 22.00) | | 0.22 (-18.68; 19.12) | | 7.60 (-15.55; 30.74) | | -5.61 (-14.93; 3.72) | |  |
| Mixed | -0.59 (-5.37; 4.19) | | 2.72 (-0.66; 6.10) | | 11.62 (7.01; 16.24) | | 1.84 (-3.16; 6.84) | | -2.56 ( -8.72; 3.61) | | -2.51 (-14.72; 9.70) | | -0.05 (-6.88; 6.78) | | 3.07 (-0.49; 6.64) | |  |
| Other | 9.46 (-3.15; 22.08) | | 4.98 (-1.02; 10.98) | | 20.00 (8.53; 31.47) | | 4.69 (-7.26;16.65) | | 1.09 (-18.92; 21.11) | | -5.90 (-26.36; 14.55) | | 11.44 (-11.84; 34.72) | | 0.60 (-11.35; 12.56) | |  |
| Socioeconomic level ^4^ |  | |  | |  | |  | |  | |  | |  | |  | |  |
| Low | 1 | | 1 | | 1 | | 1 | | 1 | | 1 | | 1 | | 1 | |  |
| Middle | 0.92 (-3.55; 5.39) | | -2.65 (-5.90; 0.60) | | -1.50 ( -6.84; 3.85) | | 4.87 (-0.04; 9.79) | | 3.84 (-2.90; 10.59) | | 0.02 (-5.66; 5.70) | | -5.88 (-10.37; -1.38) | | 2.08 (-2.58; 6.74) | |  |
| High | 7.67 (-2.78; 18.13) | | -8.34 (-14.26; -2.42) | | 0.90 (-8.84, 10.63) | | 2.81 (-7.04; 12.66) | | 1.99 (-8.67; 12.64) | | -3.66 (-12.21; 4.89) | | 2.02 (-3.65; 7.69) | | 0.56 (-7.54; 8.66) | |  |
| Education level ^5^ |  | |  | |  | |  | |  | |  | |  | |  | |  |
| Low | 1 | | 1 | | 1 | | 1 | | 1 | | 1 | | 1 | | 1 | |  |
| Middle | -1.93 (-7.41; 3.56) | | 1.06 (-2.16; 4.27) | | -0.54 (-6.56; 5.48) | | -6.28 (-11.48 ;-0.98) | | -1.16 (-10.65; 8.33) | | -4.48 (-13.02; 4.07) | | -0.14 (-4.98; 4.70) | | 3.12 (-2.19; 8.43) | |  |
| High | -9.56 (-20.68; 1.56) | | 3.86 (-2.10; 9.81) | | 0.61 (-8.31; 9.52) | | -7.53 (-14.89; -0.03) | | 6.33 (-7.38; 20.04) | | 2.77 (-8.62; 14.16) | | -1.23 (-9.41; 6.95) | | -1.35 (-5.97; 3.26) | |  |
| Transport mode ^6^ | |  | |  | |  | |  | |  | |  | |  | |  | |
| Public | | 1 | | 1 | | 1 | | 1 | | 1 | | 1 | | 1 | | 1 | |
| Private | | -1.29 (-7.09; 4.50) | | -1.12 (-5.11; 2.87) | | 0.48 (-5.70; 6.68) | | -6.91 (-12.42; -1.40) | | -2.58 (-10.09; 4.91) | | -28.33 (-77.6; 20.94) | | 0.78 (-3.91; 5.49) | | 1.76 (-2.90; 6.48) | |
| Other | | 2.71 (-4.53; 9.95) | | -0.86 (-4.92; 3.19) | | 2.00 (-5.25; 9.26) | | -0.13 (-6.44; 6.17) | | 1.76 (-7.24; 10.78) | | -0.57 (-7.79; 6.65) | | 7.74 (-1.27; 16.76) | | 6.38 (-6.84; 19.62) | |
| Public transport use ^6^ | |  | |  | |  | |  | |  | |  | |  | |  | |
| ≤ 2 days/week | | 1 | | 1 | | 1 | | 1 | | 1 | | 1 | | 1 | | 1 | |
| 3-5 days/week | | -4.02 (-9,91; 1.86) | | -1.40 (-5.53; 2.71) | | -1.82 (-7.94; 4.30) | | -5.36 (-11.75; 1.02) | | 5.88 (-3.32; 15.09) | | 13.83 (5.76; 21.90) | | -2.10 (-7.29; 3.09) | | 5.60 (2.60; 8.60) | |
| ≥ 6 days/week | | -0.52 (-5.84; 4.79) | | -1.47 (-4.90; 1.95) | | 1.52 (-3.98; 7.03) | | -3.09 (-8.12; 1.92) | | 10.48 (3.75; 17.21) | | -1.03 (-10.94; 8.86) | | 1.07 (-3.77; 5.92) | | 10.75 (4.29; 17.21) | |
| Private transport use ^6^ | |  | |  | |  | |  | |  | |  | |  | |  | |
| ≤ 2 days/week | | 1 | | 1 | | 1 | | 1 | | 1 | | 1 | | 1 | | 1 | |
| 3-5 days/week | | 1.24 (-5.31; 7.81) | | -5.60 (-10.31; -0.89) | | 0.91 (-6.07; 7.90) | | 2.07 (-5.74; 9.88) | | -0.43 (-10.58; 9.70) | | -1.03 (-10.94; 8.86) | | -4.36 (-10.86; 2.13) | | 7.27 (-0.64; 15.20) | |
| ≥ 6 days/week | | -4.69 (-9.98; 0.59) | | -3.62 (-6.92; -0.32) | | -4.88 (-10.54; 0.76) | | -6.34 (-11.38; -1.30) | | 0.12 (-6.60; 6.85) | | 5.06 (-5.39; 15.52) | | -4.25 (-8.79; 0.29) | | -4.19 (-9.72; 1.34) | |

Multilevel linear regression models, including region and cities as random effects:

^1^ Adjustment: age, ethnicity, socioeconomic and education level;

^2^ Adjustment: sex, ethnicity, socioeconomic and education level;

^3^ Adjustment: sex, age, socioeconomic and education level;

^4^ Adjustment: sex, age, ethnicity, and education level;

^5^ Adjustment: sex, age, ethnicity, and socioeconomic level;

^6^ Adjustment: sex, age, ethnicity, socioeconomic, and education level;

CI: confidence interval.

Other ethnicity (Asian, Indigenous, Gypsy, and other).

**Table S2** Multilevel linear regression models (*b* coefficient (95% CI)) between sociodemographic inequities and cycling for active transportation by country.

| Sociodemographic  inequities | Argentina | Brazil | Chile | Colombia | Costa Rica | Ecuador | Peru | Venezuela |
| --- | --- | --- | --- | --- | --- | --- | --- | --- |
| Sex ^1^ |  |  |  |  |  |  |  |  |
| Women | 1 | 1 | 1 | 1 | 1 | 1 | 1 | 1 |
| Men | 4.30 (2.08; 6.51) | 5.96 (4.28; 7.64) | 6.05 (2.35; 9.76) | 6.15 (3.87; 8.43) | 5.73 (2.65; 8.82) | 3.09 (1.67; 4.52) | 1.54 (0.55; 2.52) | 1.63 (0.43; 2.84) |
| Age group ^2^ |  |  |  |  |  |  |  |  |
| 50-65 years | 1 | 1 | 1 | 1 | 1 | 1 | 1 | 1 |
| 30-49 years | -2.99 (-5.83; -0.16) | -0.32 (-2.56; 1.92) | -1.24 (-6.17; 3.69) | 0.28 (-2.73; 3.29) | -0.32 (-4.59; 3.95) | 0.08 (-1.98; 2.14) | -1.31 (-2.75; 0.12) | -0.13 (-1.86; 1.60) |
| 18-30 years | -1.99 (-4.93; 0.95) | 2.05 (-0.30; 4.40) | -2.02 (-6.96; 2.91) | -0.32(-3.33; 2.68) | 0.37 (-3.88; 4.63) | -0.58 (-2.64; 1.49) | -0.76 (-2.16; 0.65) | -0.76 (-2.47; 0.95) |
| Ethnicity ^3^ |  |  |  |  |  |  |  |  |
| Caucasian | 1 | 1 | 1 | 1 | 1 | 1 | 1 | 1 |
| Black | 233.57 (196.06; 271.07) | 1.26 (-1.00; 3.52) | - | 2.57 (-2.01; 7.15) | -2.93 (-14.05; 8.19) | -0.30 (-5.37; 4.76) | -0.64 (-6.59; 5.31) | -0.20 (-3.35; 2.96) |
| Mixed | 0.22 (-2.31; 2.75) | 1.98 (0.01; 3.96) | 1.16 (-2.68; 5.00) | -0.94 (-3.61; 1.73) | 0.17 (-2.97; 3.30) | 1.76 (-1.53; 5.05) | 0.95 (-0.82; 2.71) | 0.08 (-1.18; 1.34) |
| Other | -1.12 (-7.89; 5.64) | 3.25 (-0.25; 6.75) | 7.12 (-2.32; 16.56) | -2.45 (-8.70; 3.80) | 3.69 (-6.48; 13.85) | -0.45 (-6.06; 5.15) | 1.13 (-4.54; 6.80) | -0.87 (-5.06; 3.32) |
| Socioeconomic level ^4^ |  |  |  |  |  |  |  |  |
| Low | 1 | 1 | 1 | 1 | 1 | 1 | 1 | 1 |
| Middle | -1.61 (-3.96; 0.75) | -2.31 (-4.22; -0.41) | 0.43 (-4.02; 4.88) | -0.46 (-3.08; 2.17) | -0.38 (-3.80; 3.05) | -0.99 (-2.56; 0.57) | 0.31 (-0.85; 1.47) | -0.43 (-2.08; 1.22) |
| High | 5.45 (-0.03; 10.92) | -2.32 (-5.80; 1.15) | -2.21 (-10.32; 5.89) | 2.15 (-3.04; 7.33) | -4.17 (-9.61; 1.27) | 0.37 (-2.00; 2.74) | 1.06 (-0.40; 2.52) | -0.28 (-3.06; 2.49) |
| Education level ^5^ |  |  |  |  |  |  |  |  |
| Low | 1 | 1 | 1 | 1 | 1 | 1 | 1 | 1 |
| Middle | 1.09 (-1.82; 3.99) | -2.48 (-4.37; -0.59) | 0.80 (-4.21 5.80) | -0.24 (-3.08; 2.60) | -1.31 (-6.11; 3.49) | -0.90 (-3.27; 1.46) | 0.08 (-1.16; 1.33) | -1.06 (-2.95; 0.83) |
| High | -2.39 (-8.20; 3.42) | -0.14 (-3.63; 3.34) | 4.66 (-2.78; 12.10) | -1.48 (-5.46; 2.50) | -1.49 (-8.55; 5.57) | -1.03 (-4.10; 2.04) | -0.80 (-2.90; 1.30) | -0.69 (-2.32; 0.94) |
| Transport mode ^6^ |  |  |  |  |  |  |  |  |
| Public | 1 | 1 | 1 | 1 | 1 | 1 | 1 | 1 |
| Private | -3.61 (-6.73; -0.49) | 0.39 (-1.93; 6.67) | -0.71 (-5.65; 4.22) | 0.22 (-2.69; 3.13) | -3.29 (-7.06; 0.48) | -3.25 (-11.50; 5.00) | 1.23 (0.02; 2.45) | -0.04 (-4.12; 4.11) |
| Other | 16.08 (12.17; 19.99) | 4.30 (1.93; 6.67) | 19.24 (13.42; 25.05) | 9.07 (5.72; 12.41) | 6.73 (2.19; 11.28) | -1.14 (-2.61; 0.32) | 2.26 (-0.03; 4.57) | -0.02 (-4.14; 4.09) |
| Public transport use^6^ |  |  |  |  |  |  |  |  |
| ≤ 2 days/week | 1 | 1 | 1 | 1 | 1 | 1 | 1 | 1 |
| 3-5 days/week | -1.52 (-4.69; 1.65) | 0.44 (-1.95; 2.85) | -4.78 (-9.67; 0.10) | 0.22 (-2.69; 3.13) | 0.73 (-3.92; 5.39) | -1.14 (-2.61; 0.32) | 1.66 (0.33; 2.99) | -2.32 (-4.23; -0.41) |
| ≥ 6 days/week | -3.42 (-6.28; -0.56) | -0.16 (-2.17; 1.83) | -3.95 (-8.34; 0.43) | 9.07 (5.72; 12.41) | 0.84 (-2.55; 4.23) | -1.69 (-3.33; -0.06) | 0.17 (-1.07; 1.41) | -2.41 (-4.40; -0.42) |
| Private transport use^6^ |  |  |  |  |  |  |  |  |
| ≤ 2 days/week | 1 | 1 | 1 | 1 | 1 | 1 | 1 | 1 |
| 3-5 days/week | -0.46 (-3.99; 3.06) | -3.29 (-6.04; -0.54) | -3.77 (-9.36; 1.80) | 1.09 (-3.03; 5.22) | -0.27 (-5.41; 4.86) | -1.38 (-3.39; 0.62) | -0.97 (-2.64; 0.68) | -0.84 (-3.31; 1.62) |
| ≥ 6 days/week | 0.38 (-2.46; 3.24) | -2.26 (-4.19; -0.33) | -4.55 (-9.05; -0.04) | -1.56 (-4.23; 1.10) | -1.56 (-4.96; 1.82) | -1.48 (-3.57; 0.60) | -0.31 (-1.48; 0.85) | -0.70 (-2.42; 1.00) |

Multilevel linear regression models, including region and cities as random effects:

^1^ Adjustment: age, ethnicity, socioeconomic and education level;

^2^ Adjustment: sex, ethnicity, socioeconomic and education level;

^3^ Adjustment: sex, age, socioeconomic and education level;

^4^ Adjustment: sex, age, ethnicity, and education level;

^5^ Adjustment: sex, age, ethnicity, and socioeconomic level;

^6^ Adjustment: sex, age, ethnicity, socioeconomic, and education level;

CI: confidence interval.

Other ethnicity (Asian, Indigenous, Gypsy, and other).

**Table S3** Multilevel linear regression models (*b* coefficient (95% CI)) between sociodemographic inequities and total active transportation by country.

| Sociodemographic  inequities | Argentina | Brazil | Chile | Colombia | Costa Rica | Ecuador | Peru | Venezuela |
| --- | --- | --- | --- | --- | --- | --- | --- | --- |
| Sex ^1^ |  |  |  |  |  |  |  |  |
| Women | 1 | 1 | 1 | 1 | 1 | 1 | 1 | 1 |
| Men | 0.07 (-4.87; 5.00) | 9.58 (6.11; 13.06) | 9.76 (3.65; 15.87) | 8.86 (3.81; 13.91) | 5.85 (-1.10; 12.79) | 9.32 (3.86; 14.78) | 1.93 (-2.09; 5.94) | -1.59 (-5.26; 2.08) |
| Age group ^2^ |  |  |  |  |  |  |  |  |
| 50-65 years | 1 | 1 | 1 | 1 | 1 | 1 | 1 | 1 |
| 30-49 years | -7.78 (-14.10; -1.47) | 0.43 (-4.21; 5.06) | -2.37 (-10.49; 5.76) | 1.89 (-4.78; 8.56) | 9.80 (0.15; 19.45) | -3.45 (-11.38; 4.47) | -3.23 (-9.04; 2.59) | -0.64 (-5.87; 4.60) |
| 18-30 years | -5.46 (-12.00, 1.09) | 2.30 (-2.56; 7.16) | -0.13 (-8.28; 8.01) | -0.53 (-7.19; 6.14) | 9.05 (-0.57; 18.67) | -8.74 (-16.66; -0.81) | -4.14 (-9.84; 1.56) | 0.62 (-4.57; 5.81) |
| Ethnicity ^3^ |  |  |  |  |  |  |  |  |
| Caucasian | 1 | 1 | 1 | 1 | 1 | 1 | 1 | 1 |
| Black | 335.42 (252.31; 418.53) | 5.52 (0.86; 10.18) | - | 5.31 (-4.79; 15.42) | -3.07 (-28.00; 21.85) | -0.53 (-20.52; 19.45) | 6.97 (-17.13; 31.07) | -5.71 (-15.71; 4.30) |
| Mixed | -0.91 (-6.54; 4.71) | 4.80 (0.70; 8.90) | 12.83 (6.49; 19.17) | 0.81 (-5.10; 6.72) | -2.52( -9.57; 4.53) | -0.60 (-13.52; 12.31) | 1.14 (-6.01; 8.28) | 3.16 (-0.68; 6.99) |
| Other | 8.41 (-6.58; 23.40) | 8.05 (0.80; 15.30) | 27.30 (11.57; 43.04) | 2.62 (-11.47; 16.70) | 5.08 (-17.71; 27.87) | -6.49 (-28.12; 15.14) | 11.07 (-13.17; 35.31) | -0.37 (-13.20; 12.47) |
| Socioeconomic level ^4^ |  |  |  |  |  |  |  |  |
| Low | 1 | 1 | 1 | 1 | 1 | 1 | 1 | 1 |
| Middle | -1.02 (-6.27; 4.24) | -5.26 (-9.21; -1.31) | -0.83 (-8.17; 6.51) | 4.52 (-1.28; 10.33) | 2.85 (-4.85; 10.56) | -1.22 (-7.24; 4.79) | -5.42 (-10.11; -0.72) | 1.63 (-3.37; 6.63) |
| High | 12.03 (-0.22; 24.27) | -10.94 (-18.10; -3.78) | -1.18 (-14.53; 12.17) | 5.40 (-6.20; 17.00) | -2.87 (-15.08; 9.33) | -2.72 (-11.76; 6.33) | 3.11 (-2.80; 9.02) | 0.17 (-8.53; 8.87) |
| Education level ^5^ |  |  |  |  |  |  |  |  |
| Low | 1 | 1 | 1 | 1 | 1 | 1 | 1 | 1 |
| Middle | -0.72 (-7.18; 5.75) | -1.72 (-5.62; 2.19) | 0.69 (-7.57; 8.95) | -6.66 (-12.93; -0.39) | -2.10 (-12.92; 8.72) | -5.32 (-14.35; 3.71) | -0.24 (-5.30; 4.82) | 2.17 (-3.56; 7.89) |
| High | -11.40 (-7.18; 5.75) | 3.59 (-3.61; 10.78) | 5.55 (-6.68; 17.78) | -9.36 (-18.20; -0.53) | 5.73 (-10.09; 21.55) | 0.13 (-11.91; 12.17) | -1.99 (-10.51; 6.53) | -2.09 (-7.04; 2.87) |
| Transport mode ^6^ |  |  |  |  |  |  |  |  |
| Public | 1 | 1 | 1 | 1 | 1 | 1 | 1 | 1 |
| Private | -4.93 (-11.83; 1.96) | -0.66 (-5.48; 4.16) | -0.26 (-8.58; 8.05) | -6.59 (-13.07; -0.12) | -5.64 (-14.18; 2.88) | -30.61 (-80.94; 19.70) | 1.91 (-3.03; 6.87) | 4.57 (-9.23; 18.43) |
| Other | 19.25 (10.65; 27.85) | 3.23 (-1.66; 8.14) | 20.82 (11.04; 30.60) | 9.09 (1.68; 16.50) | 7.90 (-2.33; 18.15) | -1.85 (-9.23; 5.52) | 10.26 (0.87; 19.66) | 6.58 (-7.24; 20.42) |
| Public transport use ^6^ |  |  |  |  |  |  |  |  |
| ≤ 2 days/week | 1 | 1 | 1 | 1 | 1 | 1 | 1 | 1 |
| 3-5 days/week | -5.25 (-12.26; 1.76) | -1.11 (-6.09; 3.85) | -6.66 (-14.89; 1.56) | -6.18 (-9.55; 2.25) | 6.13 (-4.34; 16.62) | -1.85 (-9.23; 5.52) | -0.28 (-5.71; 5.14) | -1.59 (-8.07; 4.88) |
| ≥ 6 days/week | -3.55 (-9.87; 2.76) | -1.59 (-5.74; 2.55) | -2.38 (-9.79; 5.02) | -3.64 (-9.55; 2.25) | 11.83 (4.16; 19.51) | 11.96 (3.70; 20.21) | 1.48 (-3.59; 6.56) | 8.29 (1.54; 15.05) |
| Private transport use ^6^ |  |  |  |  |  |  |  |  |
| ≤ 2 days/week | 1 | 1 | 1 | 1 | 1 | 1 | 1 | 1 |
| 3-5 days/week | 0.82 (-6.95; 8.60) | -8.95 (-14.64; -3.26) | -2.88 (-12.27; 6.49) | 3.10 (-6.07; 12.28) | -1.88 (-13.43; 9.67) | -2.18 (-12.30; 7.92) | -5.29 (-12.05; 1.46) | 6.75 (-1.60; 15.10) |
| ≥ 6 days/week | -4.95 (-11.25; 1.34) | -6.00 (-10.00; -2.01) | -9.59 (-17.19; -2.00) | -8.21 (-14.14; -2.29) | -2.20 (-9.85; 5.45) | 3.73 (-6.95; 14.41) | -4.38 (-9.14; 0.38) | -4.97 (-10.77; 0.83) |

Multilevel linear regression models, including region and cities as random effects:

^1^ Adjustment: age, ethnicity, socioeconomic and education level;

^2^ Adjustment: sex, ethnicity, socioeconomic and education level;

^3^ Adjustment: sex, age, socioeconomic and education level;

^4^ Adjustment: sex, age, ethnicity, and education level;

^5^ Adjustment: sex, age, ethnicity, and socioeconomic level;

^6^ Adjustment: sex, age, ethnicity, socioeconomic, and education level;

CI: confidence interval.

Other ethnicity (Asian, Indigenous, Gypsy, and other).
